# Analysis of Seasonal Clinical Characteristics in Patients With Bipolar or Unipolar Depression

**DOI:** 10.3389/fpsyt.2022.847485

**Published:** 2022-04-06

**Authors:** Shuqi Kong, Zhiang Niu, Dongbin Lyu, Lvchun Cui, Xiaohui Wu, Lu Yang, Hong Qiu, Wenjie Gu, Yiru Fang

**Affiliations:** ^1^Clinical Research Center and Division of Mood Disorders, Shanghai Mental Health Center, Shanghai Jiao Tong University School of Medicine, Shanghai, China; ^2^Information and Statistical Department, Shanghai Mental Health Center, Shanghai, China; ^3^Shanghai Key Laboratory of Psychotic Disorders, Shanghai, China; ^4^CAS Center for Excellence in Brain Science and Intelligence Technology, Shanghai, China

**Keywords:** bipolar disorder, depression, mixed state, biochemical, unipolar depression

## Abstract

This study was to investigate the characteristics of seasonal symptoms and non-enzymatic oxidative stress in the first hospitalized patients with bipolar and unipolar depression, aiming to differentiate bipolar depression from unipolar depression and reduce their misdiagnosis. A total of 450 patients with bipolar depression and 855 patients with depression were included in the present study. According to the season when the patients were admitted to the hospital due to the acute onset of depression, they were further divided into spring, summer, autumn and winter groups. According to the characteristics of symptoms of bipolar disorder in the DSM-5, the characteristic symptoms of bipolar disorder were collected from the medical record information, and clinical biochemical indicators that can reflect the oxidative stress were also recorded. The seasonal risk factors in patients with bipolar or unipolar depression were analyzed. The relationship of age and gender with the bipolar or unipolar depression which attacked in winter was explored. There were significant differences between groups in the melancholic features, atypical features and conjugated bilirubin in spring. In summer, there were significant differences between groups in the melancholic features, uric acid and conjugated bilirubin. In autumn, there were marked differences between groups in melancholic features, anxiety and pain, atypical features, uric acid, total bilirubin, conjugated bilirubin and albumin. In winter, the conjugated bilirubin and prealbumin were significantly different between two groups. The melancholic features and uric acid that in summer as well as melancholic features, uric acid and total bilirubin in autumn were the seasonal independent risk factors for the unipolar depression as compared to bipolar depression. In winter, significant difference was noted in the age between two groups. In conclusion, compared with patients with unipolar depression, patients with bipolar depression have seasonal characteristics. Clinical symptoms and indicators of oxidative stress may become factors for the differentiation of seasonal unipolar depression from bipolar depression. Young subjects aged 15–35 years are more likely to develop bipolar depression in winter.

## Introduction

Bipolar disorder is a mood disorder characterized by alternating or cyclic episodes of depression and mania. It is a chronic disease and requires life-long treatment, the course of disease is often prolonged and relapse is common, which cause a serious burden to the patients and their families. The clinical manifestations of bipolar disorder are more common in the stage of depression onset when the characteristic symptoms are similar to those of unipolar depression. Currently, there are no definite biomarkers of bipolar disorder and thus it is difficult to differentiate bipolar disorder from depression (1). Of note, about 50–70% of patients who are initially diagnosed with depression will be finally diagnosed as having bipolar disorder after further examinations (2, 3).

The clinical manifestations of bipolar disorder are complex and its clinical symptoms are usually changeable. There is evidence showing that the onset of bipolar disorder is closely related to season (4, 5). According to diagnostic criteria for bipolar disorder and related explanations in the DSM-5, the occurrence and remission of major depression in bipolar disorder patients may be present in a specific period of the year. Clinical experience indicates that bipolar disorder often attacks in autumn and winter, but is improved in spring in most patients. In addition, the age of patients with bipolar disorder may be a predictor of seasonal onset of bipolar disorder (6). However, the clinical evidence supporting above findings is still lacking. In addition to the typical mood disorders in the episode of mania and depression, patients with bipolar disorder often have concomitant anxiety, psychotic symptoms, and atypical characteristics. In clinical practice, above symptoms are also observed in patients with depression alone (7). However, it is unclear whether the characteristics of seasonal symptoms are different between patients with bipolar depression and unipolar depression in different seasons. In addition, some studies have shown that there are seasonal differences in the indicators of non-enzymatic oxidative stress in patients with affective disorder in the episode of depression (8). This study aimed to explore the seasonal characteristics of symptoms and the non-enzymatic oxidative stress between patients with bipolar depression and unipolar depression who were hospitalized for the first time, which may provide evidence for the differential diagnosis of unipolar depression and bipolar depression and is also helpful for the preparation for the seasonal onset and clinical characteristics of these patients and for the clinical diagnosis and treatment of these diseases.

## Subjects and Methods

### Subjects

Patients (*n* = 1,305) with bipolar disorder or depressive disorder who were hospitalized for the first time in the Mental Health Center of Medical College of Shanghai Jiaotong University between January 2009 and December 2018 were included into present study. There were 450 patients with bipolar depression (bipolar depression group) and 855 patients with severe depression (unipolar depression group). The inclusion criteria were as follows: (1) Bipolar depression and its subtypes were diagnosed according to the F31.3, F31.4, and F31.5 criteria in the ICD-10; depressive disorder and its subtypes were diagnosed according to the F32 criteria in the ICD-10 (the confirmed diagnosis requires the psychiatric examinations by three doctors, including an attending psychiatrist and a deputy director or chief psychiatrist); (2) The medical records were available; (3) The non-enzymatic oxidative stress was assesed for the first time after admission (including uric acid, total bilirubin, conjugated bilirubin, prealbumin and albumin within 15 days after first admission), and they did not use drugs that may interfere with oxidative stress before admission; (4) Patients were hospitalized for the first time; (5) Patients were 15–75 years old; (6) Both male and female patients were included. The exclusion criteria were as follows: (1) After diagnostic assessment, the diagnosis of bipolar depression, depressive disorder and their subtypes was excluded; (2) The medical records were unavailable; (3) One or more indicators reflecting non-enzymatic oxidative stress measured for the first time after admission were missing.

All the data in this study were collected retrospectively from the electronic medical records. This study was approved by the Ethics Committee of our hospital (No: 2019-15R). In order to ensure the patients' privacy, all the data related to the personal information were not described and all the investigators who had access to the data of this study signed the confidentiality agreement.

### Methods

#### Study Design and Grouping

This study was a cross-sectional study. Patients who meet the diagnostic criteria for bipolar depression or unipolar depression were included by attending psychiatrists or higher-level psychiatrists. Patients were divided into different groups according to the season when they were admitted due to acute attack of the disease. China is located in the temperate zone of the Northern Hemisphere, June when the sunshine time is the longest serves as the center of the first half year, and then spring and summer are obtained; and December when the sunshine time is the shortest serves as the center of the second half year, and then autumn and winter are obtained. Therefore, four seasons were included as a cycle. In the present study, the spring included March, April and May, the summer included June, July and August, the autumn included September, October and November and the winter included December, January and February (9). According to the above criteria, patients were divided into spring group (patients with bipolar depression and those with unipolar depression), summer group, autumn group, and winter group. The characteristics of symptoms and oxidative stress indicators were compared between patients with unipolar depression and patients with bipolar depression in different seasons. Moreover, the risk factors of bipolar depression were screened in these patients. Among patients with depression in winter group, the correlations of age and gender with season were further assessed.

### Data Collection

General demographic data included age, gender, course of disease (in months), therapeutic outcome of current treatment, and biochemical indicators related to oxidative stress (uric acid, UA; total bilirubin, T-BIL; direct bilirubin, D-BIL; prealbumin, PA; albumin, ALB). The biochemical detections were performed in the Department of Clinical Laboratory, Shanghai Mental Health Center. In addition, patients were divided into three groups according to the age: young adults (15–35 years), adults (35–55 years), and the elderly (>55 years) (10). Patients' data were collected from the hospital information system (HIS). Then, above according to diagnostic criteria for bipolar disorder and depressive disorder in the DSM-5, the characteristics of symptoms were defined in these patients. The characteristics shared in two groups included six dimensions: anxiety and pain, mixed characteristics, depression characteristics, atypical characteristics, psychotic symptoms and severity.

### Statistical Analysis

Statistical analysis was performed with SPSS version 21.0. The categorical data were compared with Chi square test between two groups or Fisher exact test if necessary. Continuous data with normal distribution are expressed as mean ± standard deviation and compared with one way analysis of variance. Continuous data without normal distribution were compared with rank sum test. Bipolar depression served as a dependent variable, and variables with significant difference between two groups were included in binomial Logistic regression analysis for the analysis of risk factors (Enter method, α_in_ = 0.05, α_out_ = 0.10). A value of *P* < 0.05 was considered statistically significant.

## Results

### Comparison of General Characteristics of Patients With Bipolar and Unipolar Depression

According to the inclusion and exclusion criteria, 1,305 patients with bipolar depression and unipolar depression were included into present, including 450 patients with bipolar disorder and 855 patients with depression. The age, gender, course of disease and outcomes after current treatment are shown in Table 1. There were significant differences in the age, gender, course of disease and outcomes after current treatment between patients with bipolar depression and those with unipolar depression.

**Table 1:** General characteristics of patients with bipolar depression and patients with unipolar depression.

**Variables**	**Bipolar** **depression**	**Unipolar** **depression**	**χ2**	***P***
*N*	450	855		
Age (yr)			92.826	0
15–35	254	269		
35–55	124	264		
>55	72	322		
Gender			16.861	0
Male	228	332		
Female	222	523		
Course of disease (month)	88.32 ± 111.42	103.18 ± 104.63	0.668	0.02
Outcomes			137.172	0
Improvement	110	284		
Others	3	17		
No change	16	49		
Significant	116	307		
Recovery	146	66		
Death	1	0		

### Symptom Characteristics and Oxidative Stress Indicators in Patients With Seasonal Bipolar Depression and Patients With Unipolar Depression

In the winter group, there were significant differences between two subgroups in the melancholic features (χ^2^ = 15.568, *P* = 0), atypical features (χ^2^ = 7.277, *P* = 0.007) and direct bilirubin (*F* = 44.73, *P* = 0). In the summer group, significant differences were noted between two subgroups in the melancholic characteristics (χ^2^ = 12.826, *P* = 0), uric acid (*F* = 1.312, *P* = 0.001) and direct bilirubin (*F* = 29.345, *P* = 0). In the autumn group, there were marked differences between subgroups in the melancholic features (χ^2^ = 30.897, *P* = 0), anxiety and pain (χ^2^ = 8.779, *P* = 0.03), atypical features (χ^2^ = 12.826, *P* = 0), uric acid (*F* = 8.05, *P* = 0.018), total bilirubin (*F* = 0.29, *P* = 0.04), direct bilirubin (*F* = 18.605, *P* = 0.) and albumin (*F* = 0.16, *P* = 0); In the winter group, significant differences were observed between two subgroups in the direct bilirubin (*F* = 0.967, *P* = 0) and prealbumin (*F* = 1.379, *P* = 0.014) (Tables 2–5).

**Table 2:** Symptoms and oxidative stress related indicators in patients who developed unipolar and bipolar depression in spring.

**Variables**	**Bipolar** **depression**	**Unipolar** **depression**	**χ2/F**	***P***
*N*	124	233		
**Annotated symptoms**				
Anxiety and pain	10/8.1%	25/11.2%	0.87	0.351
Melancholic features	43/34.7%	36/16.1%	15.568	0
Atypical features	4/3.2%	0/0	7.277	0.007
Psychotic symptoms	10/8.1%	17/7.6%	0.022	0.883
Severity			2.465	0.116
Moderate	66	138		
Severe	58	85		
**Oxidative stress**				
Uric acid	318.23 ± 89.042	302.01 ± 83.796	0.31	0.109
Total bilirubin	14.067 ± 6.148	15.103 ± 7.166	1.643	0.197
Direct bilirubin	2.269 ± 1.229	4.075 ± 2.464	44.73	0
Prealbumin	276.5 ± 72.888	288.95 ± 81.881	0.448	0.18
Albumin	42.316 ± 3.641	41.624 ± 4.493	5.347	0.246

**Table 3:** Symptoms and oxidative stress related indicators in patients who developed unipolar and bipolar depression in summer.

**Variables**	**Bipolar** **depression**	**Unipolar** **depression**	**χ^2^/F**	***P***
*N*	115	260		
**Annotated symptoms**				
Anxiety and pain	20/17.4%	36/13.8%	0.789	0.374
Mixed feature	9/7.8%	15/5.8%	0.563	0.453
Melancholic features	38/33.0%	43/16.5%	12.826	0
Atypical features	1/0.9%	0/0	2.267	0.132
Psychotic symptoms	10/8.7%	18/6.9%	0.363	0.547
Severity			3.538	0.06
Moderate	57/49.6%	156/60%		
Severe	58/50.4%	104/40%		
**Oxidative stress**				
Uric acid	332.07 ± 109.073	294.94 ± 91.01	1.312	0.001
Total bilirubin	15.468 ± 8.464	14.982 ± 7.025	4.324	0.906
Direct bilirubin	2.040 ± 1.269	3.801 ± 2.468	29.345	0
Prealbumin	265.09 ± 63.990	271.23 ± 97.961	12.545	0.936
Albumin	42.094 ± 4.189	41.276 ± 3.886	1.002	0.085

**Table 4:** Symptoms and oxidative stress related indicators in patients who developed unipolar and bipolar depression in autumn.

**Variables**	**Bipolar** **depression**	**Unipolar** **depression**	**χ^2^/F**	***P***
*N*	122	211		
**Annotated symptoms**				
Anxiety and pain	6/4.9%	27/12.8%	5.374	0.02
Mixed feature	11/9%	12/5.7%	1.333	0.248
Melancholic features	52/42.6%	32/15.2%	30.897	0
Atypical features	5/4.1%	0/0	8.779	0.03
Psychotic symptoms	6/4.9%	15/7.1%	0.628	0.428
Severity			1.177	0.278
Moderate	66	127		
Severe	56	84		
**Oxidative stress**				
Uric acid	327.36 ± 105.542	293.88 ± 86.227	8.05	0.018
Total bilirubin	14.928 ± 6.472	13.375 ± 6.030	0.29	0.04
Direct bilirubin	2.188 ± 1.177	3.808 ± 1.908	18.605	0
Prealbumin	259.93 ± 59.63	282.69 ± 73.397	5.388	0.08
Albumin	42.69 ± 4.017	40.856 ± 4.297	0.16	0

**Table 5:** Symptoms and oxidative stress related indicators in patients who developed unipolar and bipolar depression in winter.

**Variables**	**Bipolar** **depression**	**Unipolar** **depression**	**χ^2^/F**	***P***
*N*	89	161		
**Annotated symptoms**				
Anxiety and pain	16/18%	26/16.1%	0.137	0.711
Mixed feature	9/10.1%	10/6.2%	1.242	0.265
Melancholic features	24/27%	28/17.4%	3.19	0.074
Atypical features	0/89	0/68	0	/
Psychotic symptoms	12/13.5%	16/9.9%	0.724	0.395
**Severity**				
Moderate	43/48.3%	85/52.8%	0.46	0.487
Severe	46/51.7%	76/47.2%		
**Oxidative stress**				
Uric acid	318.37 ± 102.067	293.01 ± 83.196	6.926	0.091
Total bilirubin	13.019 ± 5.536	13.202 ± 5.816	0.049	0.815
Direct bilirubin	2.107 ± 1.853	3.871 ± 2.499	0.967	0
Prealbumin	262.02 ± 76.8	290.33 ± 87.293	1.379	0.014
Albumin	42.1 ± 4.5	41.049 ± 3.983	0.307	0.067

### Relationships of Age and Gender With Unipolar and Bipolar Depression in Patients Developed the Disease in Winter

Among patients who developed the disease in winter, no difference was noted in the gender, but there was marked difference in the age (*F* = 20.939, *P* = 0) between patients with unipolar depression and those with bipolar depression. In winter, the risk for bipolar depression was higher than for unipolar depression in subjects aged 15–35 years as compared to the subjects aged 35–55 years and those aged >55 years (Tables 6, 7).

**Table 6:** Correlations of age and gender with unipolar and bipolar depression in patients who developed the disease in winter.

**Variables**	**Bipolar** **depression**	**Unipolar** **depression**	**χ^2^ value**	***P*-value**
*N*	89	161		
**Gender**				
Male	43/48.3%	66/41%	1.249	0.264
Female	46/51.7%	95/59%		
**Age**				
13–35	49/55.1%	49/30.4%	20.939	0
35–55	29/32.6%	53/32.9%		
>55	11/12.4%	59/36.6%		

**Table 7:** Correlation of age with bipolar and unipolar depression in patients who developed the disease in winter (binomial logistic regression analysis).

**Variables**	**B**	**Sx**	**χ^2^**	***P***	**OR**	**95% CI**
Adults vs. young	−1.68	0.386	18.976	0	0.186	0.088–0.397
Elderly vs. young	−1.077	0.402	7.19	0.007	0.341	0.155–0.748

### Seasonal Risk Factors of Bipolar vs. Unipolar Depression

Univariate analysis showed that there were significant differences in some variables between patients with bipolar depression and those with unipolar depression in different seasons. These variables with significant difference between groups were included into binomial Logistics regression analysis. Results showed the melancholic features (OR = 2.898, *P* = 0.001) and uric acid (OR = 1.006, *P* = 0) in summer as well as melancholic features (OR = 5.035, *P* = 0), uric acid (OR = 1.006, *P* = 0.002) and total bilirubin (OR = 1.201, *P* = 0) in autumn were independent seasonal risk factors for the unipolar depression as compared to bipolar depression (Table 8).

**Table 8:** Seasonal risk factors of bipolar depression as compared to unipolar depression (binomial logistic regression analysis).

**Variables**	**B**	**Sx**	**χ^2^**	***P***	**OR**	**95%CI**
Spring direct bilirubin	−0.521	0.085	37.469	0	0.594	0.503–0.702
Atypical features	−21.781	23138.677	0	0.999	0	/
Melancholic features	−0.956	0.303	9.49	0.002	0.384	0.212–0.696
**Summer**
Melancholic features	1.064	0.316	1.324	0.001	2.898	1.559–5.385
Uric acid	0.006	0.002	17.575	0	1.006	1.003–1.009
Direct bilirubin	−0.613	0.098	39.357	0	0.542	0.447–0.652
**Autumn**
Anxiety and pain	−0.391	0.64	0.373	0.541	0.676	0.193–2.372
Melancholic features	1.616	0.397	16.607	0	5.035	2.314–10.956
Atypical features	18.46	19,982.096	0	0.999	1.04	0
Uric acid	0.006	0.002	9.526	0.002	1.006	1.002–1.01
Total bilirubin	0.183	0.038	23.081	0	1.201	1.114–1.294
Direct bilirubin	−1.306	0.173	56.71	0	0.271	0.193–0.381
Albumin	0.034	0.044	0.623	0.43	1.035	0.95–1.127
**Winter**
Direct bilirubin	−0.55	0.109	25.556	0	0.577	0.466–0.714
Prealbumin	0	0.002	0.001	0.977	1	0.996–1.004

## Discussion

It is difficult to distinguish bipolar depression from unipolar depression because they have similar clinical symptoms. Misdiagnosis may occur if the clinician fails to inquire patients who have a history of mania or mild mania, which thus may cause the prescription of antidepressants for patients with bipolar depression, increasing the risk of polar conversion and therefore delaying or even aggravating the disease condition. In clinical practice, patients are usually diagnosed according to the ICD-10 in China, but the descriptions in the ICD-10 about the depressive symptoms of bipolar disorder and depression are relatively casual. However, the diagnostic criteria in the DSM-5 systematically standardize the symptoms, and therefore are widely used in high-quality clinical studies. In addition, DSM-5 annotates the depressive symptoms of patients, and thus it can better describe the whole picture of patients with depression. Of note, the descriptions about unipolar depression and bipolar depression are similar in DSM-5. Except for the characteristics of rapid circulation, there is no difference between unipolar depression and bipolar depression. Nevertheless, unipolar depression and bipolar depression are two different entities. Available studies have revealed that the clinical symptoms of unipolar depression and bipolar depression are actually different in the stage of depression attack, and there may be seasonal differences in these symptoms (5). DSM-5 states that young people have a higher risk for depression in winter. The present study explored the seasonal risk factors of bipolar depression as compared to unipolar depression. Our results showed the presence of melancholic features in patients who developed the disease in summer and winter was a risk factor for bipolar depression. The melancholic features are mainly characterized by the loss of pleasure in all activities and the loss of response to normal happiness stimuli, or simultaneously include three other depression-related clinical symptoms (11). This annotation indicates that this depressive episode is more serious and more risky than the general depressive episode.

Our study reminds clinicians that, in case of affective disorders with depression as the main symptom in summer and autumn, the presence of melancholic features may be indicative of bipolar depression. In addition, DSM and clinical studies have indicated that age and gender are related to the occurrence of unipolar depression and bipolar depression. Thus, the associations of age and gender with unipolar and bipolar depression were further investigated in these patients. Our results showed that among the patients who developed the disease in winter, the subjects aged 15–35 years had a higher risk for bipolar depression than for unipolar depression as compared to subjects aged 35–55 years and those older than 55 years. This indicates that young patients who develop depression in winter are highly suspected of bipolar depression, but the adults and elderly patients who develop depression in winter are suspected of unipolar depression, which are consistent with the descriptions in DSM and clinical experience (12–14).

In addition to the characteristics of clinical symptoms, some studies have suggested that indicators of non-enzymatic oxidative stress may also have seasonal differences in patients with affective disorder. In recent years, numerous studies have revealed the role of oxidative stress in the pathogenesis of affective disorders. Especially in bipolar affective disorder, studies mainly focus on the enzymatic metabolites related to oxidative stress (15). On the other hand, non-enzymatic oxidative stress products such as uric acid, bilirubin, prealbumin and albumin can also reflect the level of oxidative stress (16, 17). Moreover, these indicators are routinely measured and detection of these indicators is relatively simple. Thus, if they can be measured, they may be used to predict the outcome of affective disorder in clinical practice. In the present study, uric acid and bilirubin were found to be independent risk factors for bipolar depression which attacked in summer and autumn, which was consistent with the findings from some clinical studies. In the basic research in the field of psychiatry, no biochemical and genetic indicators have been identified as having high sensitivity and specificity in predicting the psychiatric diseases. Thus, more studies are needed to investigate the role of non-enzymatic indicators related to oxidative stress in the early prediction and identification of bipolar depression and unipolar depression. Some methods such as machine learning may be employed to develop prediction models with a series of biochemical indicators, which may be clinically applicable (18–20).

There were still limitations in the present study: (1) this study was a real-world cross-sectional study, some patients with missing data were excluded, it was only a preliminary exploration, and the accurate contributors should be confirmed in more randomized double-blind controlled trials; (2) this study was a single-center study; (3) the sample size in this retrospective study should be further expanded.

## Conclusions

Taken together, patients with bipolar depression have seasonal characteristics compared with patients with unipolar depression. The clinical symptoms (such as melancholic features) and oxidative stress related indicators (such as uric acid) may be employed to distinguish seasonal unipolar and bipolar depression. In addition, young adults aged 15–35 years are more likely to develop bipolar depression in winter. The clinicians should emphasize the early identification of bipolar depression. The seasonal symptoms and oxidative stress related indicators may help clinician to differentiate depressive disorders in patients.

## Data Availability Statement

The raw data supporting the conclusions of this article will be made available by the authors, without undue reservation.

## Ethics Statement

The studies involving human participants were reviewed and approved by Ethics Committee of our Hospital (No: 2019-15R). The patients/participants provided their written informed consent to participate in this study.

## Author Contributions

SK and ZN discovered this clinical problem, designed the work, collected and analyzed data, and completed the manuscript. DL, LC, XW, LY, HQ, and WG acquired and analyzed data. DL, LC, XW, LY, YF, HQ, and WG revised the manuscript. YF contributed to the concept and design of the work and reviewed the manuscript. All authors contributed to the article and approved the submitted version.

## Funding

This study was supported by the National Key R&D Program of China (2016YFC1307100), National Natural Science Foundation of China (81930033, 81771465), Twelfth Five-Year National Plan for Science and Technology Support (2012BAI01B04), special project for big data analysis in clinical research center of Shanghai Mental Health Center (CRC2018DSJ01-1), Shanghai Clinical Research Center for Mental Health (19MC1911100), Shanghai Municipal Important Science and Technology Specific Projects (2018SHZDZX05), and Shanghai Jiao Tong University School of Medicine High-level local colleges and universities innovation team.

## Conflict of Interest

The authors declare that the research was conducted in the absence of any commercial or financial relationships that could be construed as a potential conflict of interest.

## Publisher's Note

All claims expressed in this article are solely those of the authors and do not necessarily represent those of their affiliated organizations, or those of the publisher, the editors and the reviewers. Any product that may be evaluated in this article, or claim that may be made by its manufacturer, is not guaranteed or endorsed by the publisher.

## References

[1] Buoli M Caldiroli A Cumerlato Melter C Serati M De Nijs J Altamura AC. Biological aspects and candidate biomarkers for psychotic bipolar disorder: a systematic review. Psychiatry Clin Neurosci. (2016) 70:227–44. 10.1111/pcn.12386 26969211

[2] Fang Y Wang Z Chen J. Research status and prospects of bipolar disorder in China. Chin J Psychiatry. (2015) 48:141–6. 10.3760/cma.j.issn.1006-7884.2015.03.005

[3] Fang Y Wang Z Chen J. Pay attention to the evolution of the concept and classification of bipolar disorder. Chin J Psychiatry. (2015) 48:257–9. 10.3760/cma.j.issn.1006-7884.2015.05.001

[4] Cheng S Buckley NA Siu W Chiew AL Vecellio E Chan BS. Seasonal and temperature effect on serum lithium concentrations. Aust N Z J Psychiatry. (2020) 54:282–7. 10.1177/0004867419889160 31782314

[5] Fellinger M Waldhoer T König D Hinterbuchinger B Pruckner N Baumgartner J. Seasonality in bipolar disorder: effect of sex and age. J Affect Disord. (2019) 243:322–6. 10.1016/j.jad.2018.09.073 30261447

[6] McGuire A. Diagnostic and Statistical Manual of Mental Disorders: DSM-5. 5th ed. 10.1080/09687599.2015.1062233

[7] Wang Z Fang Y. Seek the truth about bipolar disorder through heavy fog. Chin J Psychiatry. (2018) 51:81–2. 10.3760/cma.j.issn.1006-7884.2018.02.001

[8] Niu Z Yang L Chen J Fang Y. Research progress on the relationship between non-enzymatic antioxidants and bipolar disorder. Chin J Psychiatry. (2018) 51:389–92. 10.3760/cma.j.issn.1006-7884.2018.06.011

[9] The Division of Seasons for the South China Region. Actaentiarum Naturalium Universitatis Sunyatseni (1994).

[10] Yang AC Yang CH Hong CJ Liou YJ Shia BC Peng CK. Effects of age, sex, index admission, and predominant polarity on the seasonality of acute admissions for bipolar disorder: a population-based study. Chronobiol Int. (2013) 30:478–85. 10.3109/07420528.2012.741172 23281718

[11] Roza TH Kunz M Passos IC. The melancholic omega as sign of clinical depression and Darwin's description of melancholy. Aust N Z J Psychiatry. (2020) 54:646–7. 10.1177/0004867420911448 32160756

[12] Lavretsky H Reinlieb M St Cyr N Siddarth P Ercoli LM Senturk D. Citalopram, methylphenidate, or their combination in geriatric depression: a randomized, double-blind, placebo-controlled trial. Am J Psychiatry. (2015) 172:561–9. 10.1176/appi.ajp.2014.14070889 25677354 PMC4451432

[13] Tiihonen J Tanskanen A Hoti F Vattulainen P Taipale H Mehtälä J. Pharmacological treatments and risk of readmission to hospital for unipolar depression in Finland: a nationwide cohort study. Lancet Psychiatry. (2017) 4:547–53. 10.1016/S2215-0366(17)30134-7 28578901

[14] Wang X Wang H Ye Z Ding G Li F Ma J. The neurocognitive and BDNF changes of multicomponent exercise for community-dwelling older adults with mild cognitive impairment or dementia: a systematic review and meta-analysis. Aging. (2020) 12:4907–17. 10.18632/aging.102918 32191630 PMC7138588

[15] Selek S Altindag A Saracoglu G Aksoy N. Oxidative markers of myeloperoxidase and catalase and their diagnostic performance in bipolar disorder. J Affect Disord. (2015) 181:92–5. 10.1016/j.jad.2015.03.058 25942436

[16] Bartoli F Crocamo C Dakanalis A Brosio E Miotto A Capuzzi E. Purinergic system dysfunctions in subjects with bipolar disorder: a comparative cross-sectional study. Compr Psychiatry. (2017) 73:1–6. 10.1016/j.comppsych.2016.09.011 27837679

[17] Bengesser SA Lackner N Birner A Fellendorf FT Platzer M Mitteregger A. Peripheral markers of oxidative stress and antioxidative defense in euthymia of bipolar disorder–Gender and obesity effects. J Affect Disord. (2015) 172:367–74. 10.1016/j.jad.2014.10.014 25451439

[18] Chekroud AM Zotti RJ Shehzad Z Gueorguieva R Johnson MK Trivedi MH. Cross-trial prediction of treatment outcome in depression: a machine learning approach. Lancet Psychiatry. (2016) 3:243–50. 10.1016/S2215-0366(15)00471-X 26803397

[19] Rozycki M Satterthwaite TD Koutsouleris N Erus G Doshi J Wolf DH. Multisite machine learning analysis provides a robust structural imaging signature of schizophrenia detectable across diverse patient populations and within individuals. Schizophr Bull. (2018) 44:1035–44. 10.1093/schbul/sbx137 29186619 PMC6101559

[20] Young J Kempton MJ McGuire P. Using machine learning to predict outcomes in psychosis. Lancet Psychiatry. (2016) 3:908–9. 10.1016/S2215-0366(16)30218-8 27569527

